# Symptomatic Cerebral Vasospasm Following the Resection of Petroclival Meningioma

**DOI:** 10.7759/cureus.72609

**Published:** 2024-10-29

**Authors:** Sarah Bin Abdulqader, Ohud T Alharbi, Salman Alqazlan, Gmaan Alzhrani

**Affiliations:** 1 National Neuroscience Institute, King Fahad Medical City, Riyadh, SAU

**Keywords:** cerebral vasospasm, neurosurgical complication, petroclival meningioma, skull base meningioma, vasospasm

## Abstract

Petroclival meningioma (PCM) represents a formidable challenge due to its intimate association with the brainstem, basilar artery, perforating arteries, and cranial nerves. Vasospasm is a recognized complication in neurosurgery. Its incidence following skull base surgery is unknown. Here, we present a 33-year-old woman who was diagnosed with PCM and managed surgically with the anterior transpetrosal approach. Two days postoperatively, she developed sudden confusion, blurred vision, and right-sided weakness. Cerebrovascular imaging confirmed the diagnosis of vasospasm. The patient was managed successfully with combined medical and endovascular treatment of vasospasm which resulted in significant clinical improvement. Vasospasm following skull base surgery is rare. Early recognition of such rare complication and prompt response are key to achieving excellent clinical outcomes.

## Introduction

Meningiomas are the most common benign intracranial tumors, accounting for 30% of all intracranial tumors [1]. Meningiomas of the posterior fossa, which represent 10% of all meningiomas, present surgical challenges owing to their proximity to neurovascular structures. Petroclival meningiomas (PCMs) are often difficult to be dealt with surgically given their close association with the brainstem, basilar artery, perforating arteries, and cranial nerves [2]. Microsurgical treatment is often curative with different surgical approaches depending on the tumor size, extension, relationship to the tentorium and internal auditory canal (IAC), and involvement of venous structures (vein of Labbe, superior petrosal sinus, transverse sinus, and petrosal vein) [3,4]. Although most PCMs are benign, surgical treatment is associated with significant morbidity and mortality relevant to their location and association with critical neurovascular structures.

Vasospasm is a recognized complication in neurosurgery that is mostly observed in patients with aneurysmal subarachnoid hemorrhage (aSAH). The incidence of clinical vasospasm following cranial base surgery has rarely been discussed in the literature. In this report, we describe the case of a PCM that was treated surgically utilizing the anterior transpetrosal approach and showed clinical signs of vasospasm two days after surgery.

## Case presentation

Clinical presentation

**Figure 1:**
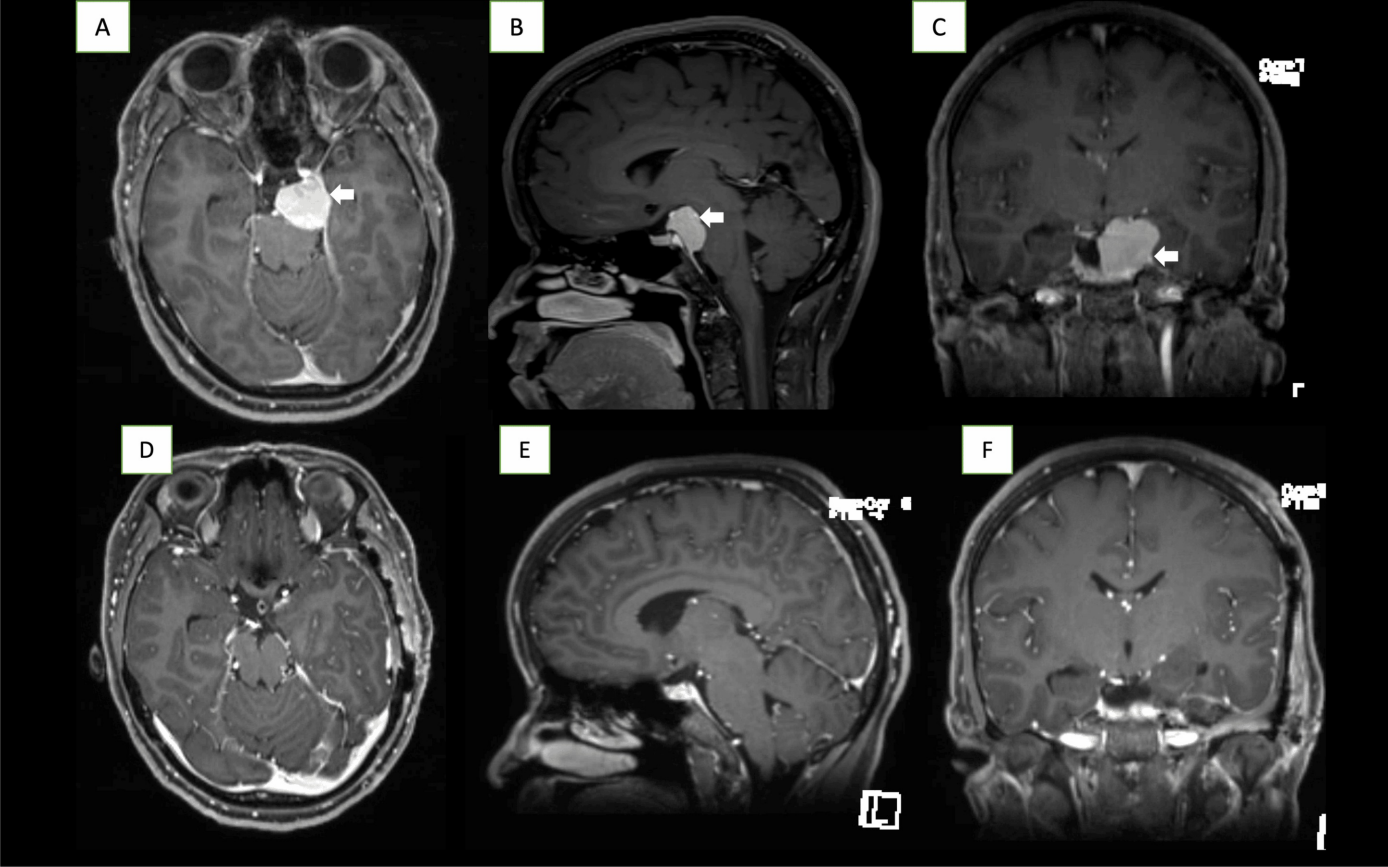
Preoperative and postoperative imaging Preoperative images A, B, and C demonstrating left petroclival meningioma with cavernous sinus extension. Postoperative images D, E, and F showing complete resection with a small residual in the cavernous sinus.

A 33-year-old woman was referred to our institution with a history of headaches, double vision, and dizziness for one year. Neurological examination showed no neurological deficits upon presentation. Neuroimaging showed a left PCM with cavernous sinus extension (Figure 1A, 1B, 1C). After counseling the patient about different management and surgical options, she was treated surgically through the anterior transpetrosal approach. A lumbar drain was inserted before starting the procedure to assist in brain relaxation during extradural dissection. The patient tolerated the procedure well and made a good recovery; however, she had trochlear nerve palsy evident immediately after surgery. Immediate postoperative imaging were satisfactory and showed complete resection of the lesion, with a small residual left at the posterior cavernous sinus (Figure 1D, 1E, 1F). The patient stayed in the neuro ICU for one day and then transferred to the ward in good condition.

**Figure 2:**
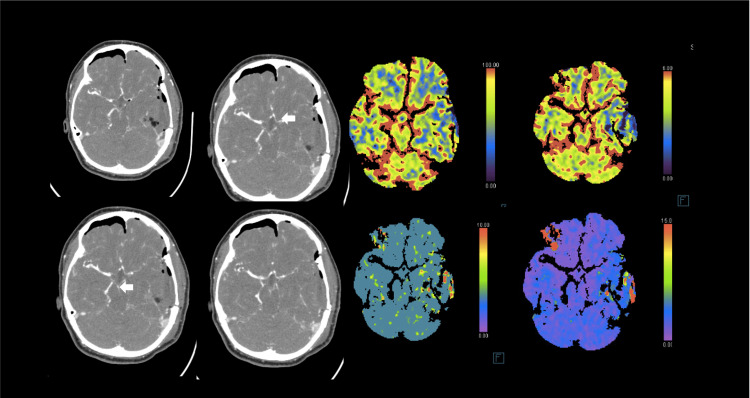
Postoperative imaging CTA demonstrating vasospasm involving the basilar artery and left internal cerebral and middle cerebral arteries that were not evident in the preoperative CTA. CT perfusion showed no evidence of core infarction or ischemic penumbra. CTA: computed tomography angiography

**Figure 3:**
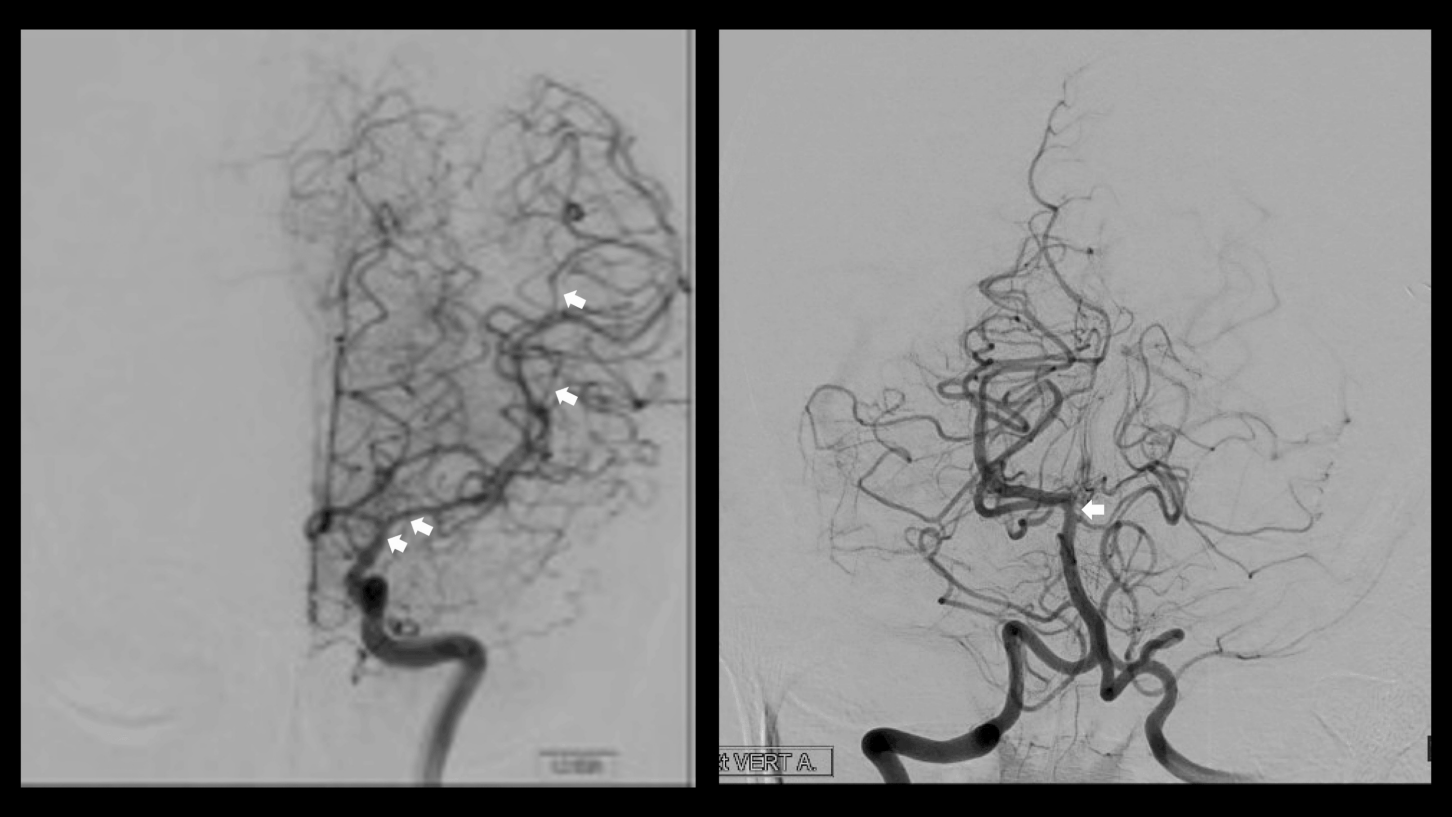
DSA DSA demonstrating vasospasm in the basilar artery and left internal cerebral and middle cerebral arteries. DSA: digital subtraction angiography

Two days postoperatively, the patient developed sudden confusion, worsening diplopia, and right-sided weakness. Her neurological examination showed confusion, left trochlear nerve palsy, and a power of 4/5 on the right side with significant pronator drift. A stroke code was activated, and imaging revealed vasospasm involving the basilar artery and left internal carotid artery (ICA) and middle cerebral artery (MCA) (Figure 2). The patient was shifted to the neuro ICU for monitoring and started on the medical treatment of vasospasm. We ensured good hydration and ruled out other possible causes of deterioration including hyponatremia, seizures, cerebral edema, or sepsis. Milrinone and phenylephrine infusion were started, to allow permissive hypertension and improve cerebral perfusion. The patient was monitored for two hours with no response to medical therapy. Therefore, digital subtraction angiography (DSA) was performed showing vasospasm involving the basilar artery and left ICA and MCA (Figure 3). Vasodilator injection using 20 mg of verapamil (10 mg was injected via the left ICA and 10 mg via the left vertebral artery) resulted in improved vessel caliber. The patient was shifted back to the neuro ICU. Medical management of vasospasm including the use of milrinone and phenylephrine infusion were continued. She showed a slow recovery over the next two days. Milrinone was tapered to discontinue over five days.

The patient was discharged home in good condition, and her weakness and consciousness level improved significantly. Her trochlear nerve palsy remained stable at the time of discharge. The residual tumor in the cavernous sinus was treated with radiation therapy resulting in good tumor control on follow-up.

At two years follow-up, the patient remained at her baseline with no neurological deficits. She underwent corrective surgery for her residual trochlear nerve palsy resulting in improved double vision and ocular motility. 

## Discussion

PCMs are defined as those meningiomas originating in the upper and middle clivus and medial to the trigeminal nerve. These meningiomas typically displace the brainstem and the basilar artery with its perforators posteriorly and contralaterally, while cranial nerves V, VII, and VIII are displaced posteriorly and laterally [5]. Different surgical approaches have been described for treating these lesions, including the retrosigmoid, transpetrosal (anterior petrosectomy, posterior petrosectomy, or a combination of both), orbitozygomatic, and endoscopic endonasal approaches [6]. The anterior transpetrosal approach is ideal for lesions that do not extend lateral and inferior to the IAC [6]. The main advantage of using this approach is the direct exposure of the posterior fossa dura at the origin of the tumor, allowing early exposure of its blood supply before tumor dissection [7]. This approach allows a wide surgical view of the trigeminal nerve area from the brainstem to Meckel's cave, the anterior aspect of the pons, and the clivus region. Therefore, major vascular structures such as the basilar artery and posterior cerebral and superior cerebellar arteries can be visualized. 

Vasospasm is a well-recognized complication in neurosurgery that has been extensively studied in the SAH literature. Its incidence following the surgical treatment of skull base tumors is unknown. An incidence of 1.9% for radiological vasospasms following the surgical resection of skull base tumors has been reported [8]. Cases of symptomatic vasospasm are limited with few cases reported including cases of pituitary adenoma, craniopharyngioma, cavernous sinus meningioma, sphenoid wing meningioma, anterior skull base meningioma, and trigeminal and vestibular schwannoma [9,10]. Various techniques were proposed to prevent vasospasm during surgery including the use of papaverine-soaked Gelfoam and intracisternal vasodilator irrigation [11]. However, their efficacies in preventing vasospasm have not been studied.

The exact pathogenesis underlying vasospasm following the resection of skull base lesions remains unclear. The proximity of PCMs to the vertebrobasilar system and its perforators may result in an indirect vessel trauma during tumor dissection, leading to vasospasm. Vessel encasement or narrowing, large tumor size, preoperative embolization, the presence of blood in the basal cistern, firm consistency, long operative time, and hyponatremia have been reported as possible risk factors for vasospasm following surgery [8,12]. In addition, vessel manipulation may stimulate the release of certain substances contributing to vessel vasoconstriction or disturbances in intracellular calcium concentrations resulting in vasospasm [13,14].

The clinical manifestations of vasospasm include neurological deficits, meningism, and altered mental status. Once the diagnosis of vasospasm is suspected, radiological imaging including computed tomography and magnetic resonance imaging should be performed to visualize the blood vessels and assess blood flow. Transcranial Doppler ultrasonography and DSA are useful for directly measuring blood flow [15].

**Figure 4:**
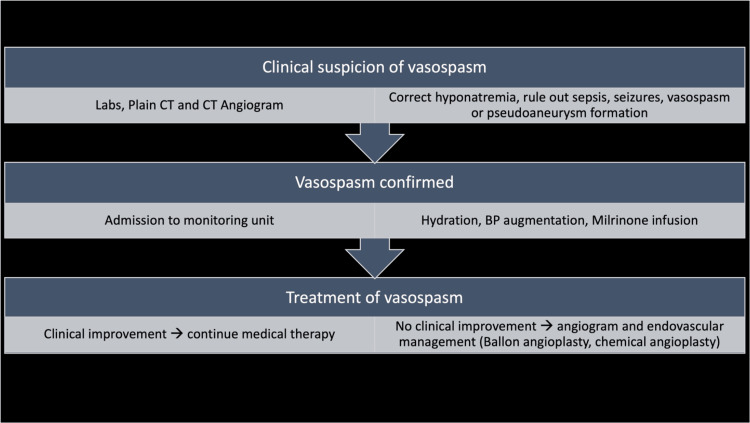
Management algorithm for suspected vasospasm following petroclival meningioma resection CT: computed tomography; BP: blood pressure

The management of vasospasm following skull base surgery is similar to that for vasospasm following aSAH. Medical management includes maintenance of euvolemia/hypervolemia, permissive hypertension, hyponatremia prevention, and seizure prevention. Endovascular treatment using balloon angioplasty, local intraarterial administration of vasodilators, or both, with the goal of restoring vessel caliber and minimizing cerebral ischemia, are necessary when there is no response to medical therapy [15] (Figure 4). Outcomes of vasospasm treatment after skull base surgery are not well documented; however, early recognition and prompt response are key to achieving excellent clinical outcomes.

## Conclusions

Vasospasm following the resection of PCM is rare. Outcomes of vasospasm treatment after skull base surgery are not well documented. However, early recognition of such rare complication and prompt response are key to achieving excellent clinical outcomes. 
